# Mitochondrial mass, a new metabolic biomarker for stem-like cancer cells: Understanding WNT/FGF-driven anabolic signaling

**DOI:** 10.18632/oncotarget.5852

**Published:** 2015-09-28

**Authors:** Rebecca Lamb, Gloria Bonuccelli, Béla Ozsvári, Maria Peiris-Pagès, Marco Fiorillo, Duncan L. Smith, Generoso Bevilacqua, Chiara Maria Mazzanti, Liam A. McDonnell, Antonio Giuseppe Naccarato, Maybo Chiu, Luke Wynne, Ubaldo E. Martinez-Outschoorn, Federica Sotgia, Michael P. Lisanti

**Affiliations:** ^1^ The Breast Cancer Now Research Unit, Institute of Cancer Sciences, University of Manchester, Manchester, UK; ^2^ The Manchester Centre for Cellular Metabolism (MCCM), Institute of Cancer Sciences, University of Manchester, Manchester, UK; ^3^ The Department of Pharmacy, Health and Nutritional Sciences, The University of Calabria, Cosenza, Italy; ^4^ The Cancer Research UK Manchester Institute, University of Manchester, Manchester, UK; ^5^ FPS - The Pisa Science Foundation, Pisa, Italy; ^6^ Department of Pathology, Pisa University Hospital, Pisa, Italy; ^7^ The Sidney Kimmel Cancer Center, Philadelphia, PA, USA

**Keywords:** mitochondria, MitoTracker, MMTV, WNT, FGF

## Abstract

Here, we developed an isogenic cell model of “stemness” to facilitate protein biomarker discovery in breast cancer. For this purpose, we used knowledge gained previously from the study of the mouse mammary tumor virus (MMTV). MMTV initiates mammary tumorigenesis in mice by promoter insertion adjacent to two main integration sites, namely Int-1 (Wnt1) and Int-2 (Fgf3), which ultimately activates Wnt/β-catenin signaling, driving the propagation of mammary cancer stem cells (CSCs). Thus, to develop a humanized model of MMTV signaling, we over-expressed WNT1 and FGF3 in MCF7 cells, an ER(+) human breast cancer cell line. We then validated that MCF7 cells over-expressing both WNT1 and FGF3 show a 3.5-fold increase in mammosphere formation, and that conditioned media from these cells is also sufficient to promote stem cell activity in untransfected parental MCF7 and T47D cells, as WNT1 and FGF3 are secreted factors. Proteomic analysis of this model system revealed the induction of i) EMT markers, ii) mitochondrial proteins, iii) glycolytic enzymes and iv) protein synthesis machinery, consistent with an anabolic CSC phenotype. MitoTracker staining validated the expected WNT1/FGF3-induced increase in mitochondrial mass and activity, which presumably reflects increased mitochondrial biogenesis. Importantly, many of the proteins that were up-regulated by WNT/FGF-signaling in MCF7 cells, were also transcriptionally over-expressed in human breast cancer cells *in vivo*, based on the bioinformatic analysis of public gene expression datasets of laser-captured patient samples. As such, this isogenic cell model should accelerate the discovery of new biomarkers to predict clinical outcome in breast cancer, facilitating the development of personalized medicine.

Finally, we used mitochondrial mass as a surrogate marker for increased mitochondrial biogenesis in untransfected MCF7 cells. As predicted, metabolic fractionation of parental MCF7 cells, via MitoTracker staining, indicated that high mitochondrial mass is a new metabolic biomarker for the enrichment of anabolic CSCs, as functionally assessed by mammosphere-forming activity. This observation has broad implications for understanding the role of mitochondrial biogenesis in the propagation of stem-like cancer cells. Technically, this general metabolic approach could be applied to any cancer type, to identify and target the mitochondrial-rich CSC population.

The implications of our work for understanding the role of mitochondrial metabolism in viral oncogenesis driven by random promoter insertions are also discussed, in the context of MMTV and ALV infections.

## INTRODUCTION

The mouse mammary tumor virus (MMTV) is a saliva- and milk-transmitted retrovirus [1-5]; however, infected mice only develop mammary tumors in adulthood [4]. This long latency period makes MMTV an interesting virus for understanding the pathogenesis of human breast cancers [6]. The provirus inserts upstream of two key integration sites, named Int-1 and Int-2 [7-10]. This process of insertional mutatgenesis is thought to be random, but involves the positive selection of genes that will ultimately provide an increase in “stemness”, a cellular growth-advantage, or perhaps both. MMTV tumors are oligo-clonal, suggesting that there is some synergy between these two different integration sites. These mammary proto-oncogenes Int-1 and Int-2 have been identified as WNT1 and FGF3 [11-13], two secreted growth factors normally involved in stem cell signaling pathways.

WNT1 is the first member of the WNT gene family, which is known to be involved in cell fate determination and patterning during embryogenesis [14, 15]. FGF3 is a member of the fibroblast growth factor family, which controls cell proliferation, morphogenesis and tissue repair [16]. Interestingly, WNT1 and FGF3 converge directly upon the WNT/β-catenin signaling cascade [17, 18]. However, it remains largely unknown exactly how WNT1/FGF3 signaling induces mammary tumorigenesis.

Here, we have created a humanized model of MMTV signaling, by over-expressing WNT1 and FGF3 in human breast cancer cells, namely MCF7 cells, an ER(+) cell line. Unbiased label-free proteomic analysis of this model system reveals the induction of EMT markers, mitochondrial proteins, glycolytic enzymes and protein synthesis machinery, consistent with an anabolic CSC phenotype. The proteins that were up-regulated by WNT/FGF-signaling in MCF7 cells, were also transcriptionally over-expressed in human breast cancer cells *in vivo*. This isogenic cell model should accelerate the identification and development of new protein biomarkers to predict clinical outcomes in breast cancer patients.

Finally, we also show that mitochondrial mass is a new metabolic biomarker for anabolic CSCs, as assessed by MitoTracker vital-staining and metabolic cell fractionation by flow-cytometry.

## RESULTS

### Generating a humanized model of MMTV signaling

**Figure 1:**
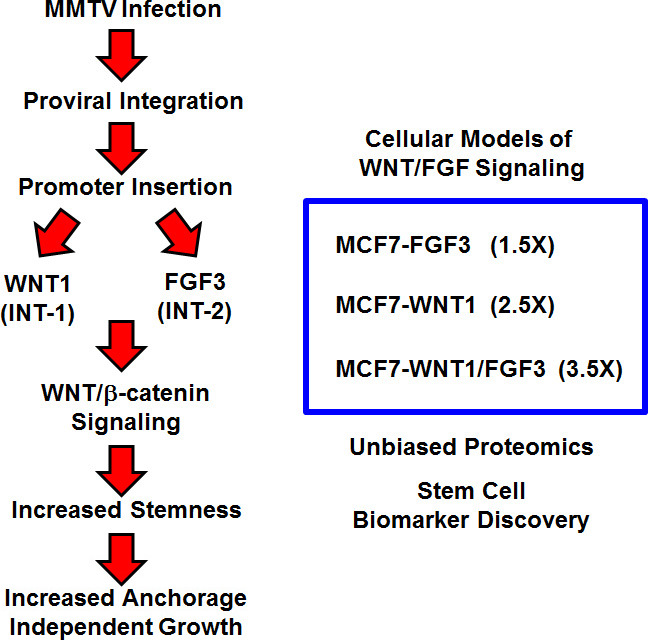
Creating a humanized experimental model for MMTV: Focus on WNT1 and FGF3 signaling To create a humanized model of MMTV signaling, we recombinantly over-expressed WNT1 and FGF3 in MCF7 cells, an ER(+) human breast cancer cell line. WNT1 and FGF3 were expressed either individually or in combination, using lenti-viral vectors carrying two different selection markers (puromycin or neomycin/G418). This isogenic cell model of “stemness” was generated to facilitate protein biomarker discovery in breast cancer, via unbiased label-free proteomics. Importantly, over-expression of FGF3, WNT1, or WNT1/FGF3 increases mammosphere formation by 1.5-, 2.5- and 3.5-fold, respectively (See Figure 2). Thus, we focused on MCF7-WNT1/FGF3 cells for further validation and proteomic analysis.

During MMTV infection of mammary epithelial cells, genomic viral integration occurs. This ultimately leads to mammary tumorigenesis in mice. Mechanistically, the MMTV virus uses a promoter insertion mechanism of mutagenesis, to drive oncogenesis [19]. More specifically, the MMTV promoter inserts upstream of two main integration sites, namely Int-1 (Wnt1) or Int-2 (Fgf3), although a few other rare integration sites have been described [11-13, 20] (Table 1). As a consequence, the MMTV promoter drives the over-expression of these secreted stem cell associated growth factors, constitutively activating Wnt/β-catenin signaling [11, 21, 22]. Thus, the MMTV model has been instrumental for understanding how “amplified” or “constitutive” stem cell signaling directly contributes to solid tumor formation (Figure 1). As such, it would be beneficial to create a humanized non-infectious model of MMTV signaling, to drive the discovery of new stem cell associated biomarkers, for predicting clinical outcome in human breast cancer patients.

**Table 1:** MMTV common proviral integration sites and gene designations

Integration site(s)	Gene Name
Int-1	Wnt-1
Int-2	Fgf-3
Int-3	Notch-4
Int-4	Wnt-3
Int-5	Aromatase; Cyp19a1
Int-6	Eukaryotic translation initiation factor 3; eIF3
Int-7	Rspo-2

Additional genes include Fgf-4, Wnt-3a and Wnt-10, among others.

Thus, in order to create a humanized model of MMTV signaling, we recombinantly over-expressed WNT1 and FGF3 in MCF7 cells, an ER(+) human breast cancer cell line. WNT1 and FGF3 were expressed either individually or in combination, using lentiviral vectors carrying two different selection markers (puromycin or neomycin/G418). For comparison purposes, empty vector controls (EV) were generated in parallel (Figure 1).

**Figure 2:**
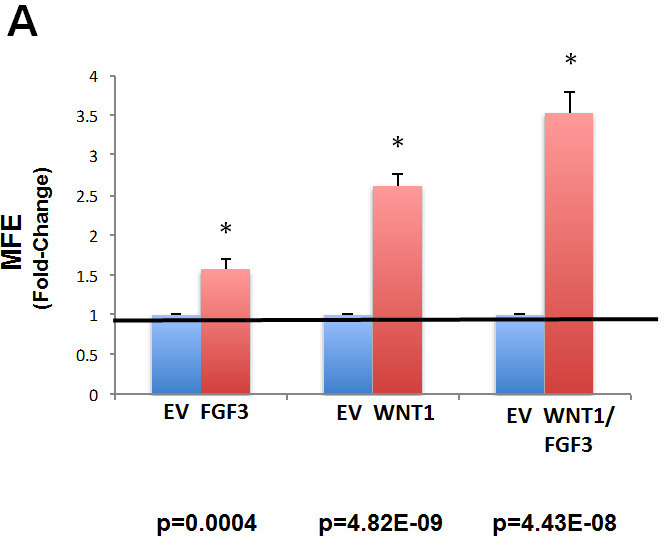

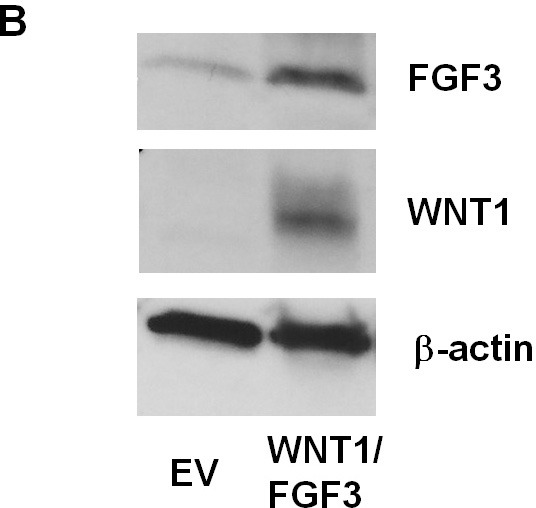
Recombinant over-expression of WNT1 and/or FGF3 in MCF7 cells significantly augments mammosphere formation **A) Mammosphere formation**. The cell lines we generated were screened for stem cell activity, using the mammosphere assay as a functional readout. Note that over-expression of either WNT1 or FGF3 significantly increased mammosphere formation by ∼1.5-fold and ∼2.5-fold, respectively. However, MCF7 cells over-expressing both WNT1 and FGF3 showed the largest increase in mammosphere formation, by up to ∼3.5-fold. Results were normalized to the three control cell lines harboring either i) EX-Neg-Lv105(puro), ii) EX-Neg-Lv151(neo) or iii) both control vectors. P-values are as indicated. Assays were performed in triplicate and repeated three times independently. MFE, mammosphere forming efficiency. **B) Immunoblot analysis**. Recombinant over-expression of WNT1 and FGF3 in these transfected cell models was validated by immunoblot analysis, with specific antibody probes. Beta-actin is shown as a control for equal loading.

Then, the various cell lines were screened for stem cell activity, using the mammosphere assay as a functional readout. Importantly, Figure 2A directly validates that over-expression of either WNT1 or FGF3 is sufficient to increase the clonal expansion of cancer stem cells. Interestingly, FGF3 and WNT1 significantly increased mammosphere formation by ∼1.5-fold and ∼2.5-fold, respectively. However, MCF7 cells over-expressing both WNT1 and FGF3 showed the largest increase in mammosphere formation, by up to ∼3.5-fold (Figure 2A).

We also validated the recombinant over-expression of WNT1 and FGF3 in these transfected cell models by immunoblot analysis, with specific antibody probes (Figure 2B).

### Conditioned media from MCF7-WNT1/FGF3 cells is sufficient to increase mammosphere formation

As WNT1 and FGF3 are secreted factors, it would be predicted that the increase in constitutive stem cell signaling could also act in a paracrine fashion on non-transfected cells. To test this hypothesis, we prepared conditioned media from MCF7-WNT1/FGF3 cells and the corresponding empty vector (MCF7-EV) control cells.

**Figure 3:**
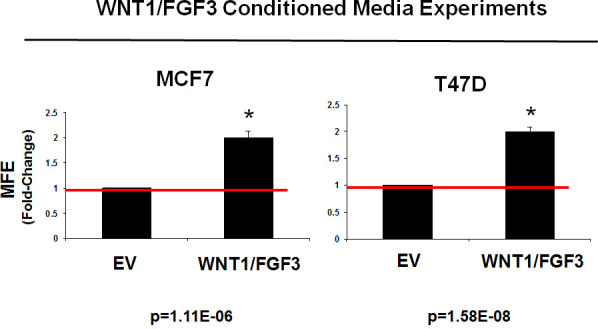
Conditioned media from WNT1/FGF3 expressing MCF7 cells increases mammosphere formation Since WNT1 and FGF3 are secreted factors, they should act in a paracrine fashion on non-transfected cells. To test this hypothesis, we prepared conditioned media from MCF7-WNT1/FGF3 cells and the corresponding empty vector (MCF7-EV) control cells. Then, we compared the ability of this conditioned media to support mammosphere formation, in untransfected parental MCF7 cells (Left panel). Note that conditioned media prepared from MCF7-WNT1/FGF3 cells significantly stimulated mammosphere formation by ∼2-fold. Nearly identical results were obtained with untransfected parental T47D cells, a second independent ER(+) breast cancer line (Right panel). Assays were performed in triplicate and repeated three times independently. MFE, mammosphere forming efficiency.

Then, we compared the ability of conditioned media to support mammosphere formation, in untransfected parental MCF7 cells. Figure 3 (Left) shows that conditioned media prepared from MCF7-WNT1/FGF3 cells significantly stimulated mammosphere formation by ∼2-fold. Importantly, virtually identical results were obtained with untransfected parental T47D cells, a second independent ER(+) breast cancer cell line (Figure 3 (Right)).

### Proteomics analysis of MCF7-WNT1/FGF3 cells reveals the upregulation of EMT markers, mitochondrial proteins, glycolytic enzymes, and the protein synthesis machinery

To better understand how WNT and FGF signaling drive the expansion of CSCs, we used unbiased label-free proteomics analysis. The proteome of MCF7-WNT1/FGF3 cells was compared to MCF7-EV control cells. We restricted our analysis to protein products that were over-expressed by >1.5-fold. Overall, our results are detailed in Tables 2, 3 and 4.

**Table 2:** Key Molecules Up-regulated by WNT1/FGF3 in MCF7 Cells: Mitochondria and Glycolysis

Symbol	Description	Fold-Change	ANOVA
**Mitochondrial-related Proteins/TCA Cycle (42)**			
ACO2	Aconitase 2, mitochondrial	Infinity	1.61E-08
IDH1	Isocitrate dehydrogenase [NADP] 1	Infinity	0.004
MDH1	Malate dehydrogenase, cytoplasmic	28.32	2.11E-05
CKMT2	Creatine kinase S-type, mitochondrial	11.04	0.015
FASN	Fatty acid synthase	7.77	0.036
CKMT1	Creatine kinase U-type, mitochondrial	7.60	2.12E-05
CKMT1B	Creatine kinase U-type, mitochondrial	6.27	5.74E-05
CKMT1A	Creatine kinase U-type, mitochondrial	5.19	7.98E-05
OAT	Ornithine aminotransferase, mitochondrial	4.79	0.0003
PC	Pyruvate carboxylase, mitochondrial	4.63	0.0001
DUT	Deoxyuridine 5′-triphosphate nucleotidohydrolase, mitochondrial	3.85	0.0001
TOMM34	Translocase of outer mitochondrial membrane 34	2.89	0.0027
GLUD2	Glutamate dehydrogenase 2, mitochondrial	2.78	0.035
MT-CO2	Cytochrome c oxidase subunit 2 (COX2)	2.66	0.0017
NQO1	NAD(P)H dehydrogenase [quinone] 1	2.60	8.31E-05
ACADVL	Very long-chain-specific acyl-CoA dehydrogenase, mitochondrial	2.46	0.0007
C21orf33	ES1 protein homolog, mitochondrial	2.37	0.01
NDUFS1	Mitochondrial NADH-ubiquinone oxidoreductase 75 kDa subunit	2.27	0.0026
SCD	Acyl-CoA desaturase	2.25	3.93E-05
GPD2	Glycerol-3-phosphate dehydrogenase, mitochondrial	2.08	0.03
HSPA9	Stress-70 protein, mitochondrial	2.07	0.0498
IDH3A	Isocitrate dehydrogenase [NAD] subunit alpha, mitochondrial	1.96	0.001
HSPD1	60 kDa heat shock protein, mitochondrial	1.95	0.02
ETFA	Electron transfer flavoprotein subunit alpha, mitochondrial	1.91	0.03
ABAT	4-aminobutyrate aminotransferase, mitochondrial	1.85	0.03
PRDX5	Peroxiredoxin-5, mitochondrial	1.83	0.037
COX4I1	Cytochrome c oxidase subunit 4 isoform 1, mitochondrial	1.83	0.007
COX6A1	Cytochrome c oxidase subunit 6A, mitochondrial	1.81	0.002
TUFM	Elongation factor Tu, mitochondrial	1.78	0.02
ATP5O	ATP synthase subunit O, mitochondrial	1.77	0.004
CLPX	ATP-dependent Clp protease ATP-binding subunit clpX-like, mitochondrial	1.72	0.016
CS	Citrate synthase, mitochondrial	1.71	0.001
ECHS1	Enoyl-CoA hydratase, mitochondrial	1.70	0.004
ATP5B	ATP synthase subunit beta, mitochondrial	1.69	0.039
PCK2	Phosphoenolpyruvate carboxykinase [GTP], mitochondrial	1.66	0.001
AK2	Adenylate kinase 2, mitochondrial	1.65	0.004
ATP5A1	ATP synthase subunit alpha, mitochondrial	1.61	0.02
ETFB	Electron-transfer-flavoprotein, beta	1.60	0.02
PRKDC	DNA-dependent protein kinase catalytic subunit (maintains mt-DNA copy number)	1.58	0.03
CHCHD2P9	Coiled-coil-helix-coiled-coil-helix domain-protein CHCHD2P9, mitochondrial	1.57	0.02
AIFM1	Apoptosis-inducing factor 1, mitochondrial	1.53	0.006
UQCRFS1P1	Putative cytochrome b-c1 complex subunit Rieske-like protein 1	1.50	0.02
**Enzymes Related to Glycolysis, the Pentose Phosphate Pathway, Glycogen, and Amino Acid Synthesis (Serine/Arginine) (14)**			
PHGDH	D-3-phosphoglycerate dehydrogenase	Infinity	2.68E-13
ASS1	Argininosuccinate synthase	17.39	3.42E-09
HK2	Hexokinase-2	11.57	3.15E-08
PKM2	Pyruvate kinase	2.83	0.003
PYGB	Glycogen phosphorylase, brain form	2.23	0.01
PFKL	6-phosphofructokinase, liver type	2.17	0.004
CAD	Glutamine-dependent carbamoyl-phosphate synthase	2.08	0.0007
PKLR	Pyruvate kinase isozymes R/L	2.08	1.46E-05
PGAM4	Probable phosphoglycerate mutase 4	1.94	0.005
G6PD	Glucose-6-phosphate 1-dehydrogenase	1.89	0.005
TKT	Transketolase	1.64	0.003
PGK2	Phosphoglycerate kinase 2	1.57	0.006
ENO1	Enolase, alpha	1.51	0.007
PGK1	Phosphoglycerate kinase	1.50	0.04

**Table 3:** Key Molecules Up-regulated by WNT1/FGF3 in MCF7 Cells: The EMT and Cell Migration

Symbol	Description	Fold-Change	ANOVA
**EMT Markers, Extracellular Matrix, Cell Migration and Cytoskeletal proteins (47)**			
MARCKS	Myristoylated alanine-rich C-kinase substrate	371.76	3.72E-05
S100A14	Protein S100-A14	96.23	0.0002
CDC42	Cell division control protein 42 homolog	68.67	5.27E-06
LGALS3BP	Galectin-3-binding protein	38.46	0.001
**FRS2**	**Fibroblast growth factor receptor substrate 2**	**11.59**	**9.69E-05**
MAST4	Microtubule-associated serine/threonine-protein kinase 4	10.81	2.62E-05
CALML5	Calmodulin-like protein 5	8.06	0.0007
CDV3	Carnitine deficiency-associated gene expressed in cardiac ventricle 3	7.67	7.58E-06
SCUBE1	Signal peptide, CUB domain, EGF-like 1	6.84	1.97E-05
S100A11	Protein S100-A11	6.66	0.026
S100A16	Protein S100-A16	6.41	0.0003
MERTK	Tyrosine-protein kinase MER	6.24	4.40E-05
NINJ1	Ninjurin-1	5.36	0.0001
TTBK2	Tau-tubulin kinase	5.31	4.15E-05
EMD	Emerin	4.38	0.0006
FLNB	Filamin-B	4.27	0.004
TTN	Titin	3.66	7.32E-05
CGNL1	Cingulin-like protein 1	3.62	0.005
TAGLN2	Transgelin-2	2.94	0.02
ACTA2	Actin, aortic smooth muscle	2.86	0.0002
TLN1	Talin-1	2.78	0.008
SEPT2	Septin-2	2.77	0.004
HMGB1	High mobility group protein B1	2.66	0.001
TPT1	Translationally-controlled tumor protein	2.54	0.028
AMOT	Angiomotin	2.40	0.008
**CTNNB1**	**Catenin, beta-1**	**2.37**	**0.0003**
TRIOBP	TRIO and F-actin-binding protein	2.35	0.003
ASAP2	Arf-GAP with SH3 domain, ANK repeat and PH domain-containing protein 2	2.23	0.01
MYH14	Myosin-14	2.20	0.02
S100A10	S100A10 protein	2.13	0.0002
TAX1BP3	Tax1-binding protein 3	2.13	0.0002
HMGB3	High mobility group protein B3	2.10	0.01
FLNA	Filamin-A	2.08	0.004
MYO18B	Myosin XVIIIB	1.99	0.0005
IQGAP1	IQ motif containing GTPase activating protein 1 (scaffold protein for CDC42)	1.98	0.026
ACTN2	Alpha-actinin-2	1.96	1.64E-05
ANXA2	Annexin A2	1.90	0.01
TAGLN3	Transgelin-3	1.89	0.01
FAM129B	Niban-like protein 1 (associated with cell invasion)	1.88	0.0008
ACTN3	Alpha-actinin-3	1.81	0.025
FAM82B	Regulator of microtubule dynamics protein 1	1.80	0.02
MYH10	Myosin, heavy polypeptide 10, non-muscle	1.79	0.04
MYOF	Myoferlin	1.60	0.0026
CAPZB	F-actin-capping protein subunit beta	1.58	0.04
MTPN	Myotrophin	1.57	0.007
TUBB2A	Tubulin beta-2A chain	1.56	0.0045
EPPK1	Epiplakin	1.51	0.0485
**Miscellaneous (17)**			
CAST	Calpastatin A	21.60	0.005
SH3BGRL	SH3 domain-binding glutamic acid-rich-like protein	14.23	0.0003
SEC24A	Protein transport protein Sec24A	12.52	7.39E-05
PABPC4	Polyadenylate-binding protein 4	6.53	0.0006
C10orf12	Uncharacterized protein C10orf12	5.71	3.46E-05
TMED4	Transmembrane emp24 domain-containing protein 4	4.99	4.34E-05
PTMS	Parathymosin	4.66	0.0001
HUWE1	E3 ubiquitin-protein ligase HUWE1	4.50	5.23E-05
PON2	Paraoxonase 2, isoform	4.28	0.003
AHNAK	Neuroblast differentiation-associated protein, AHNAK	3.68	0.0002
COMT	Soluble catechol-O-methyltransferase	3.66	0.002
STUB1	E3 ubiquitin-protein ligase CHIP	3.38	0.0001
TMEM205	Transmembrane protein 205 (chemo-resistance to cisplatin)	2.92	1.55E-05
TFF1	Trefoil factor 1	2.30	0.005
MATR3	Matrin-3	2.25	0.038
SRRM2	Serine/arginine repetitive matrix protein 2	2.19	0.03
ARF5	ADP-ribosylation factor 5	2.06	0.009

Genes encoding **FRS2** and **CTNNB1** are highlighted in **BOLD,** as they would be expected to amplify FGF and WNT signaling, respectively.

**Table 4:** Key Molecules Up-regulated by WNT1/FGF3 in MCF7 Cells: Ribosomes and Protein Synthesis

Symbol	Description	Fold-Change	ANOVA
**Ribosome-related proteins (8)**			
RPL13	60S ribosomal protein L13	6.65	7.43E-07
NPM1	NPM1 protein	3.78	0.001
RPL14	60S ribosomal protein L14	3.41	0.001
SRPRB	Signal recognition particle receptor subunit beta	2.11	0.002
RPL4	60S ribosomal protein L4	2.04	0.01
RPS5	40S ribosomal protein S5	1.99	0.006
RPL15	60S ribosomal protein L15	1.99	0.004
RPL13	60S ribosomal protein L19	1.50	0.01
**Translation initiation factors (5)**			
EIF5A	Eukaryotic translation initiation factor 5A	7.22	0.03
EIF5B	Eukaryotic translation initiation factor 5B	2.25	3.38E-06
EIF6	Eukaryotic translation initiation factor 6	2.24	0.028
EIF2S1	Eukaryotic translation initiation factor 2, subunit 1 alpha, 35kDa	1.55	0.0035
EIF3D	Eukaryotic translation initiation factor 3 subunit D	1.51	0.03
**Elongation factors (4)**			
EEF1B2	Elongation factor 1-beta	1.95	0.0498
TUFM	Elongation factor Tu, mitochondrial	1.78	0.02
EEF1D	Elongation factor 1-delta	1.70	0.03
EEF1G	Elongation factor 1-gamma	1.52	0.001
**Enzymes for tRNA synthesis (6)**			
DARS	Aspartate--tRNA ligase, cytoplasmic	3.23	0.0001
WARS	Tryptophan--tRNA ligase, cytoplasmic	2.54	0.0016
LARS	Leucine--tRNA ligase, cytoplasmic	1.90	0.002
FARSB	Phenylalanine--tRNA ligase beta subunit	1.69	0.005
EPRS	Bifunctional aminoacyl-tRNA synthetase (Glutamyl-Prolyl-tRNA Synthetase)	1.65	0.006
C22orf28	tRNA-splicing ligase RtcB homolog	1.56	0.008
**Protein folding chaperones (heat shock proteins) (14)**			
PDIA3	Protein disulfide-isomerase A3	3.59	2.39E-05
PPIB	Peptidyl-prolyl cis-trans isomerase B	3.39	1.90E-06
CALU	Calumenin	2.93	0.0002
PDIA6	Protein disulfide-isomerase A6	2.82	0.001
PDIA4	Protein disulfide-isomerase A4	2.68	0.004
HSPA1B	Heat shock 70 kDa protein 1	2.43	0.0003
HSPD1	60 kDa heat shock protein, mitochondrial	1.95	0.02
HSP90AB3P	Putative heat shock protein HSP 90-beta-3	1.61	0.026
HSPA8	Heat shock cognate 71 kDa protein	1.60	0.02
HSP90B1	Endoplasmin	1.60	0.006
HSPH1	Heat shock protein 105 kDa	1.60	0.047
PPIA	Peptidyl-prolyl cis-trans isomerase A	1.60	0.02
HSP90AB1	Heat shock protein HSP 90-beta	1.57	0.049
CANX	Calnexin	1.54	0.01
**Amino acid transport (2)**			
SLC1A5	Neutral amino acid transporter B(0)	2.77	0.0004
SLC7A5	Solute carrier family 7 (Cationic amino acid transporter, y+ system), member 5	1.66	0.04

Remarkably, >40 nuclear-encoded mitochondrial-related proteins were over-expressed in MCF7-WNT1/FGF3 cells (Table 2). Many of these proteins were related to the TCA cycle (ACO2), oxidative phosphorylation (MT-CO2), regenerating ATP (CKMT1/2) or mitochondrial biogenesis (TOMM34). In addition, MT-CO2 (a mitochondria-DNA encoded protein) was upregulated by >2.5-fold, indicative of the production of new mitochondria. In support of an anabolic phenotype, proteins related to glycolysis, the pentose phosphate pathway, glycogen metabolism and amino acid synthesis were upregulated (Table 2).

Stem-like cancer cells also undergo an epithelial-mesenchymal transition (EMT), which promotes cell migration, invasion and distant metastasis [23]. Importantly, >45 proteins known to be associated with the EMT phenotype (cell migration or invasiveness) were upregulated in MCF7-WNT1/FGF3 cells (Table 3). Examples include FRS2 (FGF receptor substrate-2; >10-fold) and β-catenin (>2-fold), which would be expected to further amplify WNT/FGF signaling, as these are down-stream elements of these convergent signaling networks. Similarly, other signaling molecules that promote the EMT and cell migration were significantly upregulated, such as MARCKS (>370-fold) and CDC42 (>65-fold).

Finally, augmented protein synthesis is another characteristic of anabolic CSCs. Note that MCF7-WNT1/FGF3 cells show the upregulation of >35 proteins related to protein synthesis (Table 4). Examples include ribosome-related proteins (RPS and RPL), translation initiation factors (EIFs), peptide elongation factors (EEFs), enzymes for tRNA synthesis, as well as chaperones for protein folding (HSPs) and amino acid transporters (SLC).

**Figure 4:**
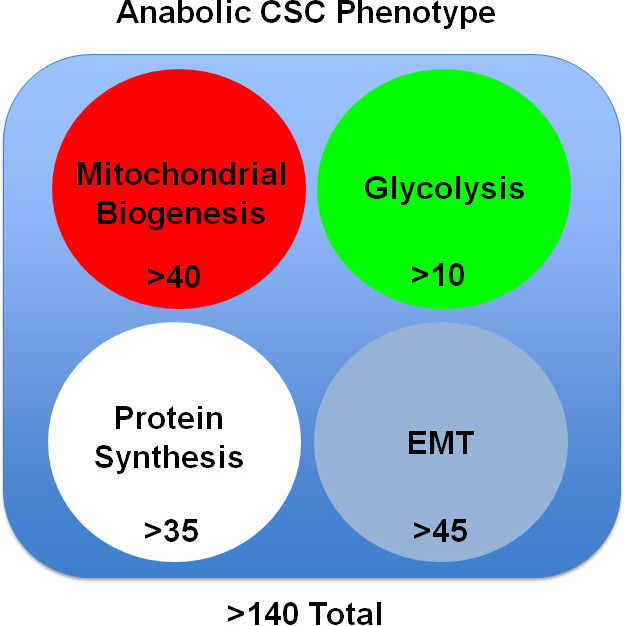
The anabolic CSC phenotype: Proteomics analysis Unbiased label-free proteomics analysis of MCF7-WNT1/FGF3 cells revealed the induction of i) mitochondrial proteins, ii) glycolytic enzymes, iii) protein synthesis machinery and iv) EMT markers, consistent with an anabolic CSC phenotype. For specific details, see Tables 2, 3 and 4. *Mitochondrial proteins –* Greater than 40 nuclear-encoded mitochondrial-related proteins were over-expressed in MCF7-WNT1/FGF3 cells. Many of these proteins were related to the TCA cycle (ACO2), oxidative phosphorylation (MT-CO2), regenerating ATP (CKMT1/2) or mitochondrial biogenesis (TOMM34). In addition, MT-CO2 (a mitochondrial DNA encoded protein) was upregulated by >2.5-fold. *Glycolytic enzymes –* More than 10 enzymes related to glycolysis, the pentose phosphate pathway, glycogen metabolism and amino acid synthesis were all upregulated in MCF7-WNT1/FGF3 cells. *Protein synthesis machinery –* Over 35 proteins related to protein synthesis, including ribosome-related proteins, enzymes for tRNA synthesis, chaperones for protein folding and amino acid transporters, were all up upregulated in MCF7-WNT1/FGF3 cells. *EMT markers –* Greater than 45 proteins known to be associated with the EMT phenotype were upregulated in MCF7-WNT1/FGF3 cells. Examples include FRS2 (FGF receptor substrate-2; >10-fold) and β-catenin (>2-fold).

Thus, MCF7-WNT1/FGF3 cells upregulate greater than 140 proteins that would be consistent with an overall anabolic phenotype (Figure 4).

### Expression of WNT1/FGF3-related targets in patient-derived human breast cancer samples

To determine the possible translational significance of our results, we intersected our WNT-FGF proteomics data with human genome-wide transcriptional profiling data. These human clinical data were derived from publically available human breast cancer samples, in which breast cancer cells were separated by laser-capture microdissection from tumor stromal cells. Transcriptional profiles were analyzed from from N=28 human breast cancer patients (See the *Materials & Methods section*). In this data set, gene expression was previously determined using Affymetrix U133A 2.0 GeneChips. A concise summary of these findings is presented in Tables 5, 6 and 7. Overall, greater than sixty WNT1/FGF3 targets (related to mitochondria, glycolysis, the EMT, and protein synthesis) that we identified in MCF7-WNT1/FGF3 cells were also transcriptionally elevated in human breast cancer cells *in vivo*. These new protein targets that we identified in MCF7-WNT1/FGF3 cells may be important for developing new strategies for the diagnosis and treatment of breast cancer.

**Table 5:** WNT1/FGF3 Targets Increased in Human Breast Cancer Cells *in Vivo*: Mitochondria and Glycolysis

Symbol	Description	Fold-Change	P-value
**Mitochondrial-related Proteins/TCA Cycle (26)**			
ATP5O	ATP synthase subunit O, mitochondrial	5.12	2.13E-06
ATP5B	ATP synthase subunit beta, mitochondrial	5.04	2.75E-06
ATP5A1	ATP synthase subunit alpha, mitochondrial	5.01	3.09E-06
COX6A1	Cytochrome c oxidase subunit 6A, mitochondrial	4.46	2.07E-05
ECHS1	Enoyl-CoA hydratase, mitochondrial	4.05	8.22E-05
MDH1	Malate dehydrogenase, cytoplasmic	3.99	9.88E-05
PCK2	Phosphoenolpyruvate carboxykinase [GTP], mitochondrial	3.88	1.43E-04
SCD	Acyl-CoA desaturase	3.70	2.55E-04
HSPA9	Stress-70 protein, mitochondrial	3.69	2.64E-04
NQO1	NAD(P)H dehydrogenase [quinone] 1	3.49	4.81E-04
HSPD1	60 kDa heat shock protein, mitochondrial	3.42	5.93E-04
COX4I1	Cytochrome c oxidase subunit 4 isoform 1, mitochondrial	3.39	6.61E-04
TUFM	Elongation factor Tu, mitochondrial	3.38	6.74E-04
C21orf33	ES1 protein homolog, mitochondrial	3.31	8.40E-04
NDUFS1	Mitochondrial NADH-ubiquinone oxidoreductase 75 kDa subunit	3.20	1.15E-03
IDH1	Isocitrate dehydrogenase [NADP] 1	3.18	1.22E-03
OAT	Ornithine aminotransferase, mitochondrial	3.17	1.25E-03
CS	Citrate synthase, mitochondrial	2.66	5.13E-03
AK2	Adenylate kinase 2, mitochondrial	2.20	1.59E-02
IDH3A	Isocitrate dehydrogenase [NAD] subunit alpha, mitochondrial	2.16	1.78E-02
PRKDC	DNA-dependent protein kinase catalytic subunit (maintains mt-DNA copy number)	2.14	1.85E-02
CLPX	ATP-dependent Clp protease ATP-binding subunit clpX-like, mitochondrial	2.11	1.96E-02
ABAT	4-aminobutyrate aminotransferase, mitochondrial	2.08	2.14E-02
ACO2	Aconitase 2, mitochondrial	1.83	3.64E-02
DUT	Deoxyuridine 5′-triphosphate nucleotidohydrolase, mitochondrial	1.87	3.37E-02
ETFA	Electron transfer flavoprotein subunit alpha, mitochondrial	1.76	4.25E-02
**Enzymes Related to Glycolysis, the Pentose Phosphate Pathway, Glycogen, and Amino Acid Synthesis (Serine/Arginine) (4)**			
PKM2	Pyruvate kinase	3.26	9.79E-04
PGK1	Phosphoglycerate kinase	2.46	8.66E-03
TKT	Transketolase	2.20	1.60E-02
ENO1	Enolase, alpha	1.96	2.75E-02

**-Transcriptional profiling data derived from the analysis of N=28 breast cancer patients are shown, high-lighting the levels of fold-upregulation observed in the epithelial cancer cell compartment (relative to the tumor stroma), and corresponding p-values derived from the analysis of these clinical samples.**

**Table 6:** WNT1/FGF3 Targets Increased in Human Breast Cancer Cells *in Vivo*: The EMT and Cell Migration

Symbol	Description	Fold-Change	P-value
**EMT Markers, Extracellular Matrix, Cell Migration and Cytoskeletal proteins (15)**			
FLNB	Filamin-B	4.81	6.21E-06
TPT1	Translationally-controlled tumor protein	3.43	5.81E-04
CDC42	Cell division control protein 42 homolog	3.11	1.48E-03
S100A11	Protein S100-A11	2.88	2.81E-03
ANXA2	Annexin A2	2.83	3.28E-03
MYOF	Myoferlin	2.67	5.00E-03
TUBB2A	Tubulin beta-2A chain	2.63	5.56E-03
SEPT2	Septin-2	2.56	6.60E-03
TAGLN2	Transgelin-2	2.42	9.47E-03
IQGAP1	IQ motif containing GTPase activating protein 1 (scaffold protein for CDC42)	2.32	1.19E-02
HMGB1	High mobility group protein B1	2.21	1.57E-02
CAPZB	F-actin-capping protein subunit beta	2.19	1.66E-02
CDV3	Carnitine deficiency-associated gene expressed in cardiac ventricle 3	2.04	2.30E-02
FAM82B	Regulator of microtubule dynamics protein 1	1.97	2.72E-02
MYH10	Myosin, heavy polypeptide 10, non-muscle	1.82	3.69E-02
**Miscellaneous (11)**			
PON2	Paraoxonase 2, isoform	4.02	9.25E-05
MATR3	Matrin-3	3.45	5.56E-04
SH3BGRL	SH3 domain-binding glutamic acid-rich-like protein	3.12	1.43E-03
AHNAK	Neuroblast differentiation-associated protein, AHNAK	2.57	6.41E-03
CAST	Calpastatin A	2.54	7.08E-03
SEC24A	Protein transport protein Sec24A	2.19	1.65E-02
PABPC4	Polyadenylate-binding protein 4	2.15	1.78E-02
COMT	Soluble catechol-O-methyltransferase	2.10	2.04E-02
STUB1	E3 ubiquitin-protein ligase CHIP	1.95	2.79E-02
TFF1	Trefoil factor 1	1.76	4.17E-02
HUWE1	E3 ubiquitin-protein ligase HUWE1	1.75	4.33E-02

**-Transcriptional profiling data derived from the analysis of N=28 breast cancer patients are shown, high-lighting the levels of fold-upregulation observed in the epithelial cancer cell compartment (relative to the tumor stroma), and corresponding p-values derived from the analysis of these clinical samples.**

**Table 7:** WNT1/FGF3 Targets Increased in Human Breast Cancer Cells *in Vivo*: Ribosomes and Protein Synthesis

Symbol	Description	Fold-Change	P-value
**Ribosome-related proteins (8)**			
SRPRB	Signal recognition particle receptor subunit beta	4.68	9.97E-06
RPL15	60S ribosomal protein L15	4.60	1.28E-05
RPL13	60S ribosomal protein L19	4.48	1.98E-05
RPL13	60S ribosomal protein L13	4.48	1.98E-05
RPL14	60S ribosomal protein L14	4.45	2.15E-05
RPS5	40S ribosomal protein S5	4.41	2.45E-05
RPL4	60S ribosomal protein L4	3.05	1.79E-03
NPM1	NPM1 protein	2.42	9.50E-03
**Translation initiation factors (3)**			
EIF2S1	Eukaryotic translation initiation factor 2, subunit 1 alpha, 35kDa	3.98	1.04E-04
EIF3D	Eukaryotic translation initiation factor 3 subunit D	2.85	3.13E-03
EIF5B	Eukaryotic translation initiation factor 5B	2.58	6.29E-03
**Elongation factors (4)**			
EEF1B2	Elongation factor 1-beta	4.08	7.56E-05
EEF1G	Elongation factor 1-gamma	3.71	2.44E-04
TUFM	Elongation factor Tu, mitochondrial	3.38	6.74E-04
EEF1D	Elongation factor 1-delta	2.50	7.67E-03
**Enzymes for tRNA synthesis (4)**			
C22orf28	tRNA-splicing ligase RtcB homolog	4.59	1.37E-05
EPRS	Bifunctional aminoacyl-tRNA synthetase (Glutamyl-Prolyl-tRNA Synthetase)	4.06	8.10E-05
DARS	Aspartate--tRNA ligase, cytoplasmic	3.43	5.87E-04
WARS	Tryptophan--tRNA ligase, cytoplasmic	2.48	8.17E-03
**Protein folding chaperones (heat shock proteins) (11)**			
HSP90AB1	Heat shock protein HSP 90-beta	4.94	4.03E-06
PPIA	Peptidyl-prolyl cis-trans isomerase A	4.29	3.74E-05
CANX	Calnexin	3.99	9.88E-05
PDIA6	Protein disulfide-isomerase A6	3.62	3.22E-04
HSPD1	60 kDa heat shock protein, mitochondrial	3.42	5.93E-04
PPIB	Peptidyl-prolyl cis-trans isomerase B	3.28	9.25E-04
HSPH1	Heat shock protein 105 kDa	3.18	1.22E-03
HSPA8	Heat shock cognate 71 kDa protein	3.11	1.49E-03
PDIA3	Protein disulfide-isomerase A3	2.53	7.22E-03
HSP90B1	Endoplasmin	2.43	9.33E-03
PDIA4	Protein disulfide-isomerase A4	2.13	1.89E-02

**-Transcriptional profiling data derived from the analysis of N=28 breast cancer patients are shown, high-lighting the levels of fold-upregulation observed in the epithelial cancer cell compartment (relative to the tumor stroma), and corresponding p-values derived from the analysis of these clinical samples.**

### MCF7-WNT1/FGF3 cells show a functional increase in mitochondrial mass and mitochondrial membrane potential

To further validate the mitochondrial phenotype of MCF7-WNT1/FGF3 cells, we used fluorescent probes to quantitate mitochondrial mass and mitochondrial membrane potential by FACS analysis. For this purpose, we used MitoTracker Deep-Red (640-nm) to measure mitochondrial mass and MitoTracker Orange (561-nm), as a measure of mitochondrial membrane potential.

**Figure 5:**
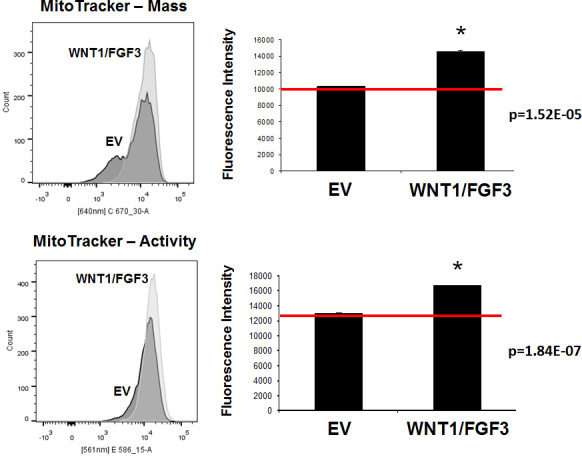
WNT1/FGF3 over-expressing MCF7 cells have increased mitochondrial mass and activity We used two different fluorescent probes to quantitate mitochondrial mass and mitochondrial membrane potential by FACS analysis. Briefly, we employed MitoTracker Deep-Red (640-nm) to measure mitochondrial mass and MitoTracker Orange (561-nm), as a measure of mitochondrial membrane potential. Note that as compared to EV control MCF7 cells, MCF7 cells overexpressing WNT1/FGF3 show a clear shift to the right, for both mitochondrial mass (Lower panels) and membrane potential (Upper panels). Quantitation of fluorescence intensity (MFI) reveals that both of these mitochondrial parameters are significantly elevated in MCF7-WNT1/FGF3 cells. P-values are as shown. These results suggest that both mitochondrial mass and function may be critical features of the CSC phenotype.

Figure 5 (Left panels) show that as compared to EV control MCF7 cells, MCF7 cells overexpressing WNT1/FGF3 show a clear shift to the right, for both mitochondrial mass and membrane potential. Furthermore, quantitation of fluorescence intensity (MFI) reveals that both of these mitochondrial parameters are significantly elevated in MCF7-WNT1/FGF3 cells (Figure 5 (Right panels)).

These results suggest that increased mitochondrial mass and function may be important features of the anabolic CSC phenotype.

### High mitochondrial mass is a key determinant of mammosphere-forming activity in parental MCF7 cells

Based on our above observations with WNT1/FGF3 signaling, we would predict that mitochondrial biogenesis is critical for mammosphere forming activity. To test this hypothesis more directly, we metabolically fractionated untransfected parental MCF7 cells, using MitoTracker Deep-Red, as a measure of mitochondrial mass. In this context, we chose to analyze three distinct metabolic phenotypic sub-groups: i) negative cells (little or no positive staining; mito-negative group); ii) bottom 5% (mito-low group); and top 5% (mito-high group). Only live cells in each group were selected for this analysis.

**Figure 6:**
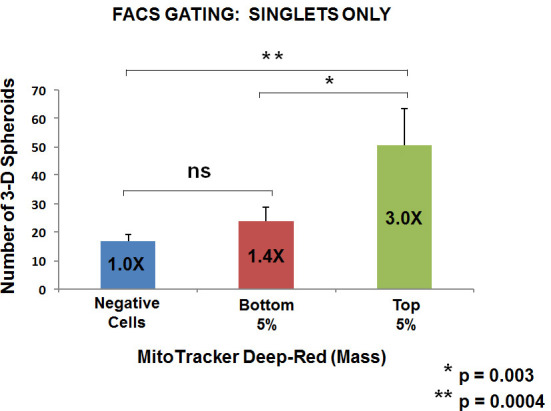
Metabolic fractionation of parental MCF7 cells directly correlates with mammosphere-forming activity: Gating for singlet cells We metabolically fractionated parental MCF7 cells, using MitoTracker Deep-Red, as a measure of mitochondrial mass. In this context, we chose to analyze three distinct metabolic phenotypic groups: i) negative cells (little or no positive staining; mito-negative group); ii) bottom 5% (mito-low group); and top 5% (mito-high group). Only live cells in each group were selected for this analysis. Five thousand live cells from each group were then seeded per well, in 6-well low attachment plates, to measure mammosphere-forming efficiency. Note that increasing mitochondrial mass results in a 3.0-fold increase in mammosphere-forming activity. Thus, the mito-deficient group showed the least sphere-forming activity, while the mito-high group showed the highest sphere-forming efficiency. Assays were performed in triplicate and repeated three times independently. The mean number of mammospheres (3-D spheroids) formed is shown.

**Figure 7:**
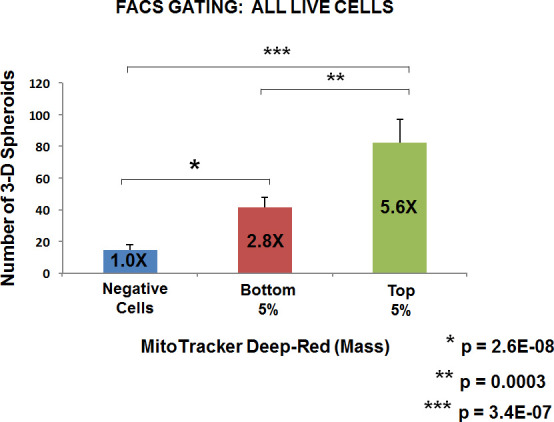
Metabolic fractionation of parental MCF7 cells directly correlates with mammosphere-forming activity: Gating for all live cells As in Figure 6, except that FACS gating included all live cells, not only live singlets. Under these conditions, note that increasing mitochondrial mass results in a >5.5-fold increase in mammosphere-forming activity. Assays were performed in triplicate and repeated three times independently. The mean number of mammospheres (3-D spheroids) formed is shown.

Five thousand live cells from each group were then seeded per well, in 6-well low attachment plates, to measure mammosphere-forming efficiency. Remarkably, Figures 6 and 7 directly show that increasing mitochondrial mass results in a 3.0 to 5.5-fold increase in mammosphere-forming activity, depending on which gating parameters are used (singlet-gating vs. all live cells). A comparison with all live cells is also shown because mammary stem cells tend to be larger than non-stem cells (See Ref # 37 for a discussion of this point).

As such, the mito-negative group showed the least 3-D sphere-forming activity, while the mito-high group showed the highest 3-D sphere-forming efficiency. Thus, we conclude that mitochondrial mass can be used to enrich for stem-like cancer cells that are able to undergo anchorage-independent propagation, under low-attachment conditions.

## DISCUSSION

**Figure 8:**
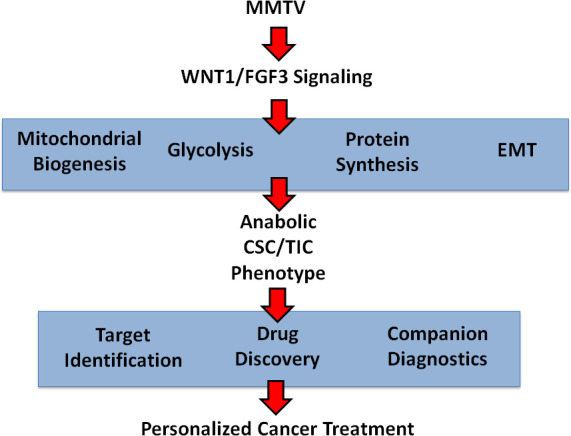
Anabolic CSC signaling: Exploiting a humanized model of MMTV signaling to identify the characteristics of anabolic CSCs and achieve the goals of personalized medicine A humanized isogenic model of MMTV-signaling was generated by co-expressing WNT1 and FGF3 in MCF7 cells, an ER(+) human breast cancer cell line. This model was first validated using the mammosphere assay to measure stem cell activity and then subjected to unbiased label-free proteomics analysis. WNT1/FGF3 protein targets identified in this manner were found to be transcriptionally over-expressed in human breast cancer cells *in vivo*, providing clinical validation of the success of our approach. Thus, we established that the anabolic CSC phenotype is characterized by the induction of EMT markers, mitochondrial proteins, glycolytic enzymes and protein synthesis machinery. These represent new classes of identified protein targets for drug discovery and the identification of companion diagnostics, to eradicate anabolic CSCs.

The mouse mammary tumor virus (MMTV) initiates mammary tumorigenesis in mice by promoter insertion adjacent to two main integration sites, namely Int-1 (Wnt1) and Int-2 (Fgf3), driving the propagation of mammary cancer stem cells [11-13, 20]. Here, we developed an isogenic cell model of MMTV signaling to facilitate protein biomarker discovery in breast cancer (Figure 8). More specifically, we over-expressed WNT1 and FGF3 in MCF7 cells, an ER(+) human breast cancer cell line. Importantly, MCF7 cells over-expressing both WNT1 and FGF3 showed a 3.5-fold increase in mammosphere formation. Proteomic analysis of this model system revealed the induction of EMT markers, mitochondrial proteins, glycolytic enzymes and protein synthesis machinery, consistent with an anabolic phenotype. The WNT1/FGF3-induced increases in mitochondrial function were validated by MitoTracker staining. Proteins up-regulated by WNT/FGF-signaling in MCF7 cells, were also transcriptionally over-expressed in breast cancer cells *in vivo*, based on the bioinformatic analysis of public datasets of laser-captured epithelial tumor cells from breast cancer patients. We believe that this isogenic cell model will accelerate the identification of new protein biomarkers to predict clinical outcomes in breast cancer patients. Remarkably, metabolic fractionation of parental MCF7 cells, via MitoTracker staining, indicated that mitochondrial mass is a key determinant of mammosphere-forming activity. The mito-negative group showed the least sphere-forming activity, while the mito-high group showed the highest sphere-forming efficiency. Thus, new mitochondrial biogenesis is critical for the successful propagation of stem-like cancer cells.

### Role of new mitochondrial biogenesis in WNT-signaling and asymmetric cell division in stem cells

Interestingly, two previous studies have also linked WNT signaling to new mitochondrial biogenesis, in the context of skeletal muscle function and osteoblastic differentiation [24, 25]. For example, Yoon et al performed an si-RNA screen to identify novel protein targets that are critical for driving mitochondrial biogenesis in skeletal muscle cells [24]. For this purpose, they screened the effects of si-RNAs on C2C12 cells, representing >6,300 genes, using a high-throughput FACS-based assay to measure mitochondrial function. Overall, they identified >150 proteins not previously recognized to be involved in the regulation of mitochondrial biogenesis. Bioinformatics analysis of this data set identified WNT/β-catenin signaling as a key regulator of mitochondrial biogenesis. This was functionally validated by using si-RNAs targeting β-catenin and Axin2. Moreover, treatment of C2C12 cells with Wnt3a increased mitochondrial biogenesis by nearly 2-fold, which also directly correlated with a functional increase in oxygen consumption. Expression of a dominant-negative form of TCF4 blocked the effects of Wnt3a on mitochondrial biogenesis, indicating that the canonical Wnt-pathway was responsible for the metabolic effects of Wnt3a. Interestingly, Wnt3a mediated mitochondrial biogenesis also appeared to be dependent on down-stream effectors, such as IRS-1 and c-MYC [24]. Therefore, the effects of WNT/β-catenin signaling on mitochondrial biogenesis, may ultimately be mediated by the c-MYC proto-oncogene.

Asymmetric cell division is required for the maintenance of the stem cell phenotype and also occurs in stem-like cancer cells. Recently, Weinberg and Sabatini assessed how mitochondria are apportioned during asymmetric cell division, using an immortalized model of mammary epithelial stem cells [26]. Interestingly, they observed that “newly-synthesized” mitochondria were concentrated in stem cells during asymmetric cell division, while “old” mitochondria were segregated into daughter cells. As such, asymmetric cell division requires new mitochondrial biogenesis, for the propagation the stem cell phenotype. These findings could mechanistically explain our current results, that high-mitochondrial mass (Figures 6 and 7) directly correlates with “stemness” and mammosphere-forming efficiency.

### Role of mitochondrial biogenesis in anchorage-independent growth, in transformed fibroblasts and CSCs

In 1984, Klebe and Harriss described the use of a colorimetric tetrazolium dye, namely MTT, to distinguish between normal and transformed fibroblasts, when the two different immortal isogenic cell lines were co-cultured [27]. More specifically, they showed that SV40-transformed BALB/3T3 fibroblasts, which were undergoing anchorage-independent growth (foci-formation), were highly MTT-positive [27]. In contrast, non-transformed quiescent BALB/3T3 cells were MTT-negative, showing little or no staining [27]. As MTT-staining has largely been attributed to mitochondrial oxidative function and redox activity, these results may be the first description of an association between anchorage-independent growth and mitochondrial function.

In accordance with these findings, Fisher et al., 2011 [28] demonstrated that PGC1-α mediated activation of mitochondrial biogenesis is indeed required for the anchorage-independent growth of RAS-transformed fibroblasts. Moreover, recent studies with XCT790, a chemical inhibitor of the ERR-α/PGC1-α signaling network, directly showed that blocking mitochondrial biogenesis is sufficient to effectively prevent the anchorage-independent survival and propagation of epithelial CSCs [29]. Quantitatively similar results were also obtained with azithromycin and doxycycline [29, 31], two well-established antibiotic inhibitors of mitochondrial biogenesis, which target mitochondrial protein translation.

Thus, our current results are also consistent with the idea that mitochondrial power somehow helps to energize anchorage-independent growth, which is a key characteristic of CSCs.

### Viral oncogenesis, promoter insertion and energy metabolism

MMTV is known to cause the development of mammary tumors by promoter insertion proximal to cellular proto-oncogenes, that when over-expressed, confer an oncogenic phenotype. This appears to be largely through the constitutive activation of WNT/β-catenin signaling. Here, we show that this signal transduction process also leads to the activation of mitochondrial biogenesis and an increase in the machinery necessary for protein synthesis, which is characteristic of an anabolic CSC phenotype. Previous studies have also shown that the ability of WNT/β-catenin signaling to increase mitochondrial biogenesis is dependent on c-MYC activation, but in the context of skeletal muscle cells [24]. In further support of these ideas, MMTV Int-6 is eukaryotic translation initiation factor 3 (eIF3), which serves as a scaffolding protein to increase protein synthesis (Table 1).

Avian leukosis virus (ALV) is another pathogen that induces cancer, via a promoter insertion-based mechanism [32, 33]. More specifically, ALV infection leads to proviral intergration and promoter insertion, driving the development of myeloid leukosis and, ultimately, frank leukemia in chickens. Interestingly, the most common ALV integration sites include c-MYC and hTERT, as well as other gene products related to mitochondrial biogenesis and function (NDUFS6 and PARK2) [34].

**Figure 9:**
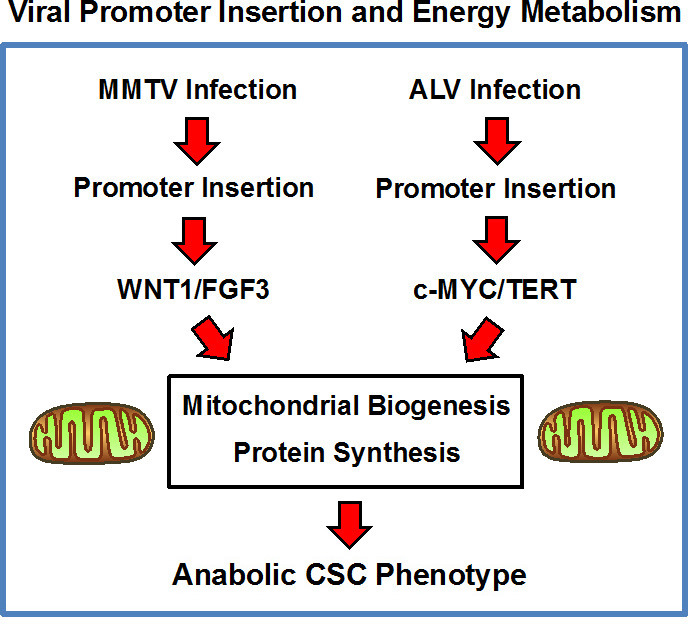
Convergent role of energy metabolism in the pathogenesis of viral oncogenesis, driven by promoter insertion: A new metabolic hypothesis We propose that MMTV and ALV may induce oncogenesis by a convergent metabolic mechanism, which relies on an anabolic CSC phenotype, characterized by increased mitochondrial biogenesis and augmented protein synthesis. See the Discussion section for further details. ALV, avian leukosis virus; MMTV, mouse mammary tumor virus.

Taken together, these data imply that MMTV and ALV may induce oncogenesis by a convergent metabolic mechanism, which relies on the down-stream activation of c-MYC, driving increased mitochondrial biogenesis. Interestingly, c-MYC is also known to increase protein synthesis, by targeting translation initiation, as well as by directly increasing ribosomal biogenesis [35, 36]. As such, MMTV and ALV may both ultimately induce the anabolic CSC phenotype, via increased mitochondrial biogenesis and increased protein synthesis (Figure 9). In further support of this idea, hTERT over-expression also appears to be directly associated with an anabolic CSC phenotype, driving increased mitochondrial biogenesis and augmented protein synthesis [37]. Thus, this intriguing hypothesis, regarding the existence of a convergent metabolic mechanism, underlying MMTV and ALV oncogenesis, undoubtedly deserves further study.

### Mitochondrial DNA content and mitochondrial mass both increase during the transition from normal tissue to hyperplasia and malignancy

Interestingly, previous studies in human endometrial cancer have monitored i) mt-DNA content (by RT-PCR) and ii) mitochondrial mass (using the enzyme activity of citrate synthase), during the transition to malignancy. More specifically, they observed that both of these parameters increased by up to 2 to 3 fold, when normal endometrial tissue was directly compared to endometrial cancer [38]. Similarly, they also observed that the protein expression levels of three mitochondrial-related transcription factors (TFAM, NRF1 and PGC1-alpha) were all significantly increased by nearly 2-fold [39]. Taken together, these results are all consistent with an increase in mitochondrial biogenesis, during the pathogenesis of tumor initiation.

Similarly, we have previously shown that markers of mitochondrial mass and mitochondrial activity are specifically localized to the basal stem cell layer in normal human mucosa, which co-localizes with Ki67, an established marker of cell proliferation [40]. In addition, this mitochondria-rich population of cells is dramatically expanded in head and neck cancers [40] and breast cancers [41-43]. Moreover, recombinant over-expression of mitochondrial-related proteins, such as PGC1-alpha/beta, POLRMT, MitoNEET or GOLPH3, is sufficient to promote tumor growth, by up to 3-fold, in xenografted pre-clinical models of human breast cancers [44, 45]. Finally, mitochondrial biogenesis and mass are also significantly increased in hematological malignancies, such as in chronic lymphocytic leukemia (CLL) and acute myeloid leukemia (AML) [46-49].

This increase in mitochondrial mass also appears to be part of a normal developmental process, as mitochondrial biogenesis increases between 25 to 50 fold, during mammalian embryogenesis, especially from the two-cell stage to the early blastocyst [50]. This early stage of embryogenesis reflects the proliferative expansion normal progenitor cells. Interestingly, pluripotent ES cell lines are derived from the inner cell mass of the blastocyst.

## CONCLUSIONS

In summary, the use of mitochondrial mass as a surrogate metabolic biomarker of mitochondrial biogenesis allows for the identification of stem-like cancer cells, facilitating CSC enrichment for future biomarker studies and aiding in the design of novel therapeutic interventions. In this context, MCF7 cells over-expressing WNT1/FGF3 will provide a novel model system for these ongoing investigations.

## MATERIALS AND METHODS

### Materials

Breast cancer cell lines (MCF7 and T47D) were originally purchased from the ATCC. Gibco-brand cell culture media (DMEM and DMEM/F12) was purchased from Life Technologies. Lentiviral vectors encoding WNT1 [EX-B0110-Lv105(puro)] and FGF3 [EX-A0154-Lv151(neo)] were obtained commercially from Genecopoeia (USA), along with appropriate empty vector controls [EX-Neg-Lv105(puro) and EX-Neg-Lv151(neo)]. Antibodies directed against FGF3 (# HPA012692, Sigma) and WNT1 (# ab15251, Abcam) were also obtained commercially. MitoTracker probes (Deep-Red and Orange) were purchased from Molecular Probes, via Life Technologies.

### MCF7 cell viral transduction and antibiotic selection

Lentiviral particles harboring human WNT1 [EX-B0110-Lv105(puro)] or human FGF3 [EX-A0154-Lv151(neo)] were prepared and used to stably transduce MCF7 cells, according to the manufacturer's protocol. After 24 hours, media containing the virus was removed and replaced with standard media. Cells were then selected with puromycin (2 μg/ml) or G418 (2 mg/ml), for up to 10 days. MCF7 cells harboring the empty vector alone controls were generated at the same time in parallel. MCF7-WNT1/FGF3 cells were generated by serial transduction with both WNT1 and FGF3 lentiviral vectors.

### WNT1 and FGF3 immunoblotting

Transduced MCF7 cells were seeded in 10 cm dishes for 72 hrs. Then, cells were lysed in RIPA buffer (Sigma), containing proteinase inhibitors (Roche) and kept at 4°C for 30 minutes. Lysates were collected by centrifugation for 10 minutes at 10,000 × g, and protein concentration were determined using the BCA protein assay kit (Pierce). Samples were diluted into SDS-PAGE sample buffer and were boiled for 5 minutes before being separated by SDS-PAGE, using a 4-15% gradient Mini-PROTEAN TGX Gel (Biorad). Samples were then transferred onto a nitrocellulose membrane (Biorad), blocked in 5% milk in TBS-Tween 20 (Sigma) and probed with antibodies directed against WNT1 or FGF3 and β-actin (Santa Cruz Biotechnology, #sc-1616), using a secondary antibody at a dilution of 1 to 5000. Bound antibodies were detected using the Supersignal West Pico Chemiluminiscent substrate (ThermoScientific). Alternatively, in the laboratory, blots were also routinely processed with a blocking solution containing BSA, as a blocking agent. Similarly, other comparable antibodies directed against β-actin were used, but were obtained from different commercial sources, such as Sigma.

### Assessment of mammosphere forming activity

Mammosphere formation was carried out, essentially as described previously by Clarke and colleagues, without any significant modifications [51]. MCF7 cells were plated at a density of 500 cells/cm^2^ in mammosphere medium in culture dishes coated with poly-HEMA (Sigma, #P3932). After 5 days, 3D spheroids with a diameter greater than 50 μm were counted using a microscope, fitted with a graticule eye-piece, and the percentage of cells which formed spheroids was calculated and normalized to one (1 = 100 % MSE; mammosphere forming efficiency). Mammosphere assays were performed in triplicate and repeated three times independently.

### Conditioned media experiments

One million MCF7 cells transfected with WNT1/FGF3 or empty vector alone controls were plated for 24 hours in DMEM (10% FCS). Cells were then washed in PBS, the subsequently cultured for 72 hours in 10 ml of DMEM/F12 phenol-red free media (mammosphere media). Media was then collected and cells were removed by centrifugation at 1800 rpm for 10 minutes. Conditioned media was then added directly to mammosphere assays of parental untransfected breast cancer cell lines (MCF7 and T47D) in a ratio of 1:1 with fresh mammosphere formation media.

### Unbiased label-free proteomics analysis

Proteomics analysis was carried out essentially as we previously described, with minor modifications [52, 53]. Statistical analyses were performed using ANOVA and only fold-changes in proteins with a p-value less than 0.05 were considered significant. Unbiased proteomics and the statistical analysis of the results were performed by the Biological Mass Spectrometry Core Facility, at the Cancer Research UK Manchester Institute, under the supervision of Dr. Duncan L. Smith.

### Bioinformatics analysis with publically available human breast cancer clinical data

To determine the possible translational significance of our proteomics analysis, we intersected our MCF-based WNT/FGF proteomics data with human genome-wide transcriptional profiling data. These human clinical data were derived from publically available human breast cancer samples, in which breast cancer cells were separated by laser-capture microdissection from tumor stromal cells. Transcriptional profiles were analyzed from N=28 human breast cancer patients [54].

### Analysis of mitochondrial mass and membrane potential

To measure mitochondrial activity, cells were stained with MitoTracker Orange (#M7510, Invitrogen), whose accumulation in mitochondria is dependent upon membrane potential. To measure mitochondrial mass, cells were stained with MitoTracker Deep Red (#M22426, Invitrogen), localizing to mitochondria regardless of mitochondrial membrane potential. Cells were incubated with pre-warmed MitoTracker staining solution (diluted in PBS/CM to a final concentration of 10 nM) for 30-60 min at 37°C. All subsequent steps were performed in the dark. Cells were washed in PBS, harvested, and re-suspended in 300 μL of PBS. Cells were then analyzed by flow cytometry. Data analysis was performed using FlowJo software.

### Metabolic fractionation of parental MCF7 cells using MitoTracker

Using MitoTracker Deep-Red staining as a marker of mitochondrial mass, we metabolically fractionated parental MCF7 cells, using FACS analysis and cell collection. In these experiments, we analyzed three different metabolic sub-groups: i) negative cells (little or no positive staining; mito-negative group); ii) bottom 5% (mito-low group); and top 5% (mito-high group). Only live cells in each group were selected for this analysis. Five thousand live cells from each group (performed in triplicate) were then seeded per well, in 6-well low attachment plates, to measure mammosphere-forming efficiency. Gating for cell size was varied to take into account the observation that stem-like mammary cells may be physically larger than “bulk” cancer cells, as was previously suggested.

This method is a further refinement of a protocol used by Farnie, Sotgia and Lisanti, in a companion study published in parallel [55]. Importantly, very similar results were obtained here, indicating that the method is operator independent. For example, see Figure 4 (Panel A) in Farnie et al., 2015, for comparison purposes [55]; however, in this companion paper, the mito-negative group was not analyzed.

### Statistical analyses

Statistical significance was determined using the Student's t-test or ANOVA, where appropriate. Values of less than 0.05 were considered significant. Data in figures are shown as the mean ± SEM, unless stated otherwise.

## SUPPLEMENTARY TABLES



## Abbreviations

CSCs: cancer stem-like cells
MMTV: mouse mammary tumor virus
TICs: tumor-initiating cells
EMT: epithelial-mesenchymal transition
ALV: avian leukosis virus
MTT: 3-(4,5-dimethylthiazol-2-yl)-2,5-diphenyltetrazolium bromide
