# Supplementary material for: Mitochondrial mass, a new metabolic biomarker for stem-like cancer cells: Understanding WNT/FGF-driven anabolic signaling
Source: Oncotarget. 2015 Sep 28;6(31):30453–71. doi: 10.18632/oncotarget.5852 (PMC4741544; doi:10.18632/oncotarget.5852)
Supplement: Supplementary file 1 [file oncotarget-06-30453-s001.pdf]

Table 1: MMTV common proviral integration sites and gene designations.

**Integration site(s)**	**Gene Name**
Int-1	Wnt-1
Int-2	Fgf-3
Int-3	Notch-4
Int-4	Wnt-3
Int-5	Aromatase; Cyp19a1
Int-6	Eukaryotic translation initiation factor 3; eIF3
Int-7	Rspo-2

Additional genes include Fgf-4, Wnt-3a and Wnt-10, among others.
